# Henoch-Schönlein Purpura With Scrotal Involvement: A Case Report and Literature Review

**DOI:** 10.1097/MPH.0000000000002161

**Published:** 2021-04-21

**Authors:** Yue Ma, Shanyun Zhang, Jiye Chen, Han Kong, Juanjuan Diao

**Affiliations:** * Shandong University of Traditional Chinese Medicine; † Department of Pediatrics, Affiliated Hospital of Shandong University of Traditional Chinese Medicine, Jinan, Shandong, People’s Republic of China

**Keywords:** Henoch-Schönlein purpura, orchitis, epididymitis, case report, literature review

## Abstract

Henoch-Schönlein purpura (HSP) is the most common vasculitis of childhood and affects the small blood vessels, leading to arthritis, abdominal pain, and renal involvement. However, scrotal involvement is a rare complication of HSP and scrotal pain. Swelling is the most frequent clinical presentation and can be easily confused with testicular torsion. If not treated in time, the scrotal inflammation will result in irreversible testicular necrosis. We report a 6-year-old male with HSP and scrotal involvement, characterized by swelling and pain on the left side of the scrotum, rashes on both lower extremities, and epididymitis. He was treated with conservative care, corticosteroids, and antibiotic therapy. We were able to avoid surgical intervention. On the 10 days of treatment, he recovered sufficiently well and was discharged. We have reviewed the literature related to HSP with scrotal involvement, identified 21 cases, and revealed that steroid therapy and/or antibiotics are the first-line of therapy in children with scrotal involvement. Vasculitis in the scrotum may predispose to testicular torsion, which is a complication that should not be overlooked. Clinicians should be aware of the atypical types of HSP. Timely diagnosis and appropriate treatment are essential for achieving the best results.

Henoch-Schönlein purpura (HSP) is the most common systemic vasculitis in childhood. The dominant clinical features are non-thrombocytopenic purpura, abdominal pain, gastrointestinal bleeding, arthritis, and renal involvement. In 2013, Trnka reviewed the literature and found that the annual incidence of HSP varies between 10 and 30 cases per 100,000 children.^1^ Most cases occur in children under 10 years of age, where males are more common by a 2:1 ratio.

The diagnosis of HSP is based on clinical findings. In 2019, the European Union Against Rheumatism and the European Pediatric Rheumatology Society developed a new classification of vasculitis in children, which has since replaced the HSP classification developed by the American Rheumatology Association in 1990,^2^,^3^ as listed in Table 1.

**TABLE 1:** Diagnostic Criteria for HSP (EULAR/PRES Unified Standard)

Obvious palpable purpura in the presence of at least one of the following:
Diffuse abdominal pain
Any biopsy showing predominant IgA deposition
Acute arthritis/joint pain
Renal involvement (hematuria and/or proteinuria)

EULAR indicates European alliance of associations for rheumatology; HSP Henoch-Schönlein purpura; Ig, immunoglobulin; PRES, paediatric rheumatology European society.

Generally, the prognosis of HSP in children is favorable. However, there are severe complications: myocarditis, mumps, and involvement of the nervous system (eg, intracranial hemorrhage, encephalopathy), respiratory system (eg, pulmonary hemorrhage), and urinary system (eg, scrotal involvement, penile involvement).

Allen et al^4^ reported the first case of HSP with scrotal involvement in 1960. HSP with scrotal involvement affects ∼2% to 38% of patients.^5^ The clinical manifestations are mostly unilateral scrotal pain, tenderness, and swelling.^6^ The patients can present with scrotal edema, pain or tenderness in the physical examination, or an imaging abnormality (eg, epididymitis-orchitis, epididymitis).^7^,^8^ Similar to intussusception in the gastrointestinal involvement in HSP, testicular vasculitis can easily induce testicular torsion. HSP with scrotal involvement must be evaluated for testicular torsion by scrotal ultrasound and radionuclide scanning.^4^

Although characteristic clinical features are well known to pediatricians, they may not be familiar with other atypical complications. Previous systematic reviews of HSP discussed the involvement of the respiratory and nervous system.^9^,^10^ However, there has not been a thorough literature review of HSP with scrotal involvement. This report presents a 6-year-old male with HSP with scrotal involvement and reviews 21 cases reported in the literature since 1986.

## CASE PRESENTATION

A previously healthy 6-year-old Chinese boy developed a skin rash in both lower extremities, followed by 2 weeks of severe abdominal pain and bloating. He was quickly treated at a local hospital and diagnosed with HSP. Laboratory data revealed that the white blood cell (WBC) was 10.74×10^9^/L, red blood cell (RBC) was 4.50×10^12^/L, platelet count (PLT) was 406×10^9^/L, C-reactive protein (CRP) was 0.83 mg/L, neutrophilic granulocyte ratio was 41.50%, lymphocyte ratio was 49.30%, d-dimer was 1.30 mg/L. Urinalysis showed urine protein and the WBC were weakly positive, as was the test for occult blood in the stool. Abdominal ultrasonography showed no apparent abnormalities in the liver, bile, pancreas, spleen, and kidney. During the hospitalization, he was successively given intravenous azithromycin, cefoperazone, phloroglucinol, Bozhi glycopeptide, omeprazole, etamsylate, human immunoglobulin, methylprednisolone, and compound amino acid for 8 days. However, the treatment was not ideal, and the child continued to experience abdominal pain, with yellow-green emesis. He was transferred to our hospital for further evaluation and management.

**FIGURE 1:**
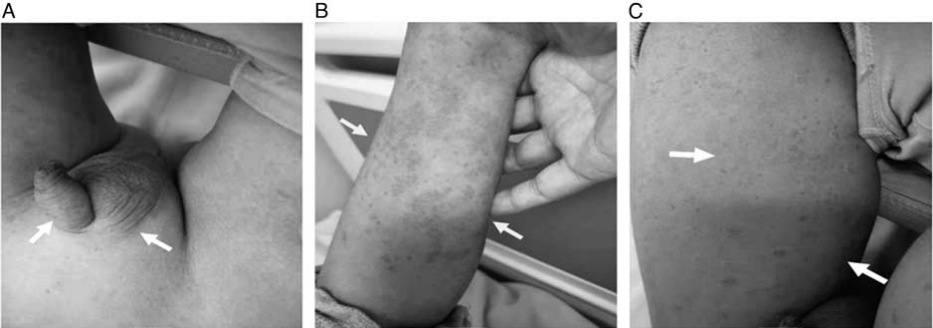
The distribution of purpura in children and clinical manifestations of scrotal involvement. A, Photograph shows the swelling of the both scrotum with a purpuric rash and the left side more severely. Purpura palpable also appeared in the penis (arrows). B, Purpura appeared on his left arm with edema and pain (arrows). C, Purpura palpable on both thighs, the rash higher than the skin (arrows).

Upon arrival, his vital signs were within the normal range. The physical examination revealed abdominal pain, and a skin rash spread diffusely over both feet, which extended to the entire body. He also had pain, skin rash on his left arm, vomiting, inability to eat, irritability, and bloody stools. Both testicles were swollen and painful with the left side more severely affected. We found no edema in the lower extremities (Fig. 1). A blood test revealed the WBC was 19.22×10^9^/L and the d-dimer was 2.32 μg/mL. Urinalysis showed that the urine WBC was 13.86 P/μL, RBC was 346.5 P/μL, urine protein was positive, and a routine stool examination showed the occult blood was positive. The prothrombin time and activated partial thromboplastin time were normal, and the serum levels of immunoglobulin (Ig)G, IgA, IgM, antinuclear antibody, and perinuclear-antineutrophil cytoplasmic antibody cytoplasmic-antineutrophil cytoplasmic antibody, antistreptolysin O, complement 3 (C3), and C4 were also within normal limits. Scrotal ultrasonography suggested left epididymitis (scrotal ultrasonography images showed the size and shape of the bilateral testicles were normal, parenchymal echo uniformity, normal blood flow signal. The left testicle volume increased, echo decreased, blood flow signal, increased. No abnormal echo in right testicle). According to the patient’s clinical presentation, he was diagnosed as HSP with epididymitis and nephritis. We initiated treatment with methylprednisolone, ceftriaxone sodium, and oral lyophilizing. During treatment, the patient was placed on a liquid diet. We performed a second scrotal ultrasonography review after 10 days of treatment (scrotal ultrasonography image showed no abnormalities in bilateral testicles and epididymis). At this point, the patient recovered well without any surgical intervention and was later discharged. We monitored the patient for changes in the rash, joint symptoms, and gastrointestinal manifestations during the period of using corticosteroids. No recurrence was over the following 3 months.

## REVIEW OF THE LITERATURE

A literature review was performed using the PUBMED database, using the terms “Henoch-Schönlein purpura” or “anaphylactoid purpura,” and “epididymitis,” “scrotal involvement,” and “children.” We selected articles from the English literature which described cases of pediatric patients with HSP and with scrotal involvement, described symptoms, diagnostic findings, treatment, and outcomes.

Between 1986 and 2020, 21 case reports of children with HSP described scrotal involvement. The main findings of these case reports are shown in Table 2.

**TABLE 2:** Published Case Reports of Henoch-Schönlein Purpura With Scrotal Involvement in Children

References	Age (d)	Time Between HSP Symptoms and Scrotal Involvement (d)	Symptoms (Scrotum)	Diagnostic Findings	Treatment and Outcome
Brodie et al^7^	4	3, after the HSP	Swelling and pain	DUS: epididymitis	Conservative treatment, antibiotics+NSAIDs. Prognosis is good
Kaminsky et al^11^	8	3, before the HSP	Swelling and pain	DUS: epididymitis	Conservative treatment, antibiotics+glucocorticoids, NSAIDs. Prognosis is good
Modi et al^6^	5	Simultaneously	Swelling and pain	DUS: epididymitis	Conservative treatment, antibiotics+corticosteroid. Prognosis is good
Güneş et al^12^	7	30, after the HSP	Swelling and pain, erythema	DUS: epididymitis	Bilateral testicular fixation, prednisolone therapy. Prognosis is good
Güneş et al^12^	6	30, after the HSP	Swelling and pain, erythema	DUS: epididymitis	Bilateral testicular fixation. Prognosis is good
Akgun^13^	7	7, before the HSP	Swelling and pain	DUS: increased blood flow in the testicles. No clear diagnosis	Surgical exploration: no testicular torsion. Prognosis is good
Palumbo^14^	6	4, before the HSP	Swelling and pain, erythema	DUS: epididymitis, scrotal nuclear scanning: no testicular torsion	Conservative treatment, dexamethasone IV+oral prednisolone. Prognosis is good
Fukuda^15^	12	2, after the HSP	Swelling and pain, erythema	DUS: epididymitis CT: necrotic testis	Surgical examination: unexplained testicular infarction
Huang et al^16^	4	3, before the HSP	Swelling and pain, erythema	Nuclear scanning: epididymitis	Conservative treatment, dexamethasone IV+oral prednisolone. Prognosis is good
Dayanir et al^17^	7	3, after the HSP	Swelling and pain, erythema	DUS: epididymitis	Conservative treatment, aspirin IV+corticosteroid therapy. Prognosis is good
Lim et al^8^	5	17, after the HSP	Swelling and pain, erythema	DUS: epididymitis	Conservative treatment, oral prednisolone. Prognosis is good
Verim et al^18^	5	1, after the HSP	Swelling and pain in the scrotum	DUS: epididymitis	Conservative treatment, antibiotic+corticosteroid therapy. Prognosis is good
Gómez Parada et al^19^	7	2, after the HSP	Swelling and pain, erythema	DUS: epididymitis	Conservative treatment, corticosteroid therapy. Prognosis is good
Gómez Parada et al^19^	4	1, after the HSP	Swelling and pain, erythema	DUS: epididymitis	Conservative treatment, corticosteroid therapy. Prognosis is good
Sakai et al^20^	8	2, after the HSP	Swelling and pain, erythema	DUS: epididymitis	Conservative treatment, corticosteroid therapy. Prognosis is good
Januário and Santiago^21^	5	Simultaneously	Swelling and pain, erythema	DUS: epididymitis	Conservative treatment, prognosis is good
Stein et al^22^	4	1, after the HSP	Enlarged left testicle, swelling and pain, erythema	DUS: epididymitis	Conservative treatment, corticosteroid therapy. Prognosis is good
Sudakoff et al^23^	3	1, after the HSP	Swelling and pain, erythema	DUS: epididymitis	Conservative treatment. Prognosis is good
Clark and Kramer^24^	3	5, before the HSP	Swelling and pain, erythema	Scrotal exploration: no testicular torsion	Conservative treatment, amoxicillin IV. Prognosis is good
Chamberlain and Greenberg^25^	6	5, after the HSP	Swelling and pain, erythema	Surgical: no testicular torsion	Conservative treatment. Methylprednisolone IV. Prognosis is good
Ben-Chaim et al^26^	3.5	4, after the HSP	Swelling and pain, erythema	DUS: epididymitis	Conservative treatment, methylprednisolone IV. Prognosis is good

CT indicates computed tomography; DUS, color Doppler ultrasound; HSP, Henoch-Schönlein purpura; IV, intravenous; NSAIDs, nonsteroidal anti-inflammatory drugs.

## CLINICAL OBSERVATIONS OF SCROTAL INVOLVEMENT

The average age at HSP onset with scrotal involvement was 5.69±2.12 years. Almost all children with scrotum involvement had scrotal pain with redness and swelling without difficulty in urination. In the case review, we found that 10 (48%) patients had gastrointestinal involvement (eg, abdominal pain or vomiting).^7^,^8^,^11^,^12^,^15^,^17^,^19^,^25^ Four (19%) patients had fever,^6^,^16^,^22^,^26^ 9 (42%) patients had joint involvement,^7^,^13^–^16^,^21^–^23^,^25^ 2 (9%) patients had penis involvement,^7^,^8^ and hematuria was found in 2 (9%) cases.^11^,^12^ Regarding the onset of HSP and scrotal involvement, 14 cases of scrotal involvement manifested after the onset of HSP (67%),^7^,^8^,^12^,^15^,^17^–^20^,^22^,^23^,^25^,^26^ 5 cases were before the onset of HSP (24%),^11^,^13^,^14^,^16^,^24^ and 2 cases occurred simultaneously with HSP (9%).^5^,^21^ It shows that scrotal involvement can occur at any point in relation to the diagnosis of HSP. How scrotal, joint, renal, and gastrointestinal involvement impact each other is unclear and needs further investigation. Tabel and colleagues, showed that scrotal involvement is related to renal involvement. In contrast, Ha and colleagues believed that scrotal involvement had no connection with the renal involvement and that the occurrence time of these complications with HSP is not absolute. We need to understand the complications of HSP more and to make a correct diagnosis.

## LABORATORY ANALYSIS ACCORDING TO SCROTAL INVOLVEMENT

Among the 21 patients with scrotal involvement, the imaging studies performed that 14 patients showed epididymitis^7^,^8^,^11^,^12^,^18^–^20^,^23^,^25^; 1 patient had orchitis^24^ and patients had epididymo-orchitis.^6^,^13^,^17^,^21^–^23^ Surgical exploration revealed that epididymal and scrotal hyperemia, and epididymis without torsion occurred in 4 patients.^12^,^13^,^25^ One patient had a testicular infarction due to an early misdiagnosis of epididymitis and conservative treatment. One patient was quickly treated with surgery after diagnosis^15^ and 1 patient had urethral calculi in the late stage.^11^ The examinations of epididymitis and orchitis are mostly physical examination and ultrasound examination. Owing to the influence of infectious factors, WBC, CRP, and erythrocyte sedimentation rate are normal or higher than normal. Ha and colleagues found that serum C3 in the scrotum is high for patients with scrotal involvement. Serum C4, CH50, IgA, IgG, IgM, IgE, ASO, RF, and antinuclear antibody are not significantly associated with findings of scrotal involvement.^27^

## TREATMENT AND OUTCOME OF SCROTAL INVOLVEMENT

Most patients with HSP involving the scrotum received conservative treatment. Eleven patients received steroids,^8^,^12^,^14^,^16^,^17^,^19^,^20^,^22^,^25^,^26^ 2 patients received antibiotics,^7^,^24^ and 3 patients received both.^6^,^11^,^18^ The remaining case reports did not mention medication.

Five patients received a nonconservative treatment, among them, 1 patient underwent surgical treatment due to testicular necrosis.^15^ The final 4 received surgical exploration because they could not rule out testicular torsion.^12^,^13^,^25^

## DISCUSSION

HSP was renamed to IgA vasculitis in the 2012 International Classification of Vasculitis. IgA-predominant immune deposits are occasionally found in testicular blood vessels so that the testis can also be regarded as the target organ of this systemic vasculitis; Zhao et al^28^ confirmed this view. Various studies have reported different incidence of scrotal involvement in HSP cases. Weber and colleagues reported that boys with HSP were up to 24%, and the incidence of unilateralism was up to 60%. Chao and colleagues reported that 10% of acute scrotum at presentation. Ha and Lee reported 26 of 120 boys (21.7%) were diagnosed with HSP and scrotal involvement.^29^,^30^

In this literature review, 90% of the children initially developed skin symptoms. However, the scrotum may be the first symptom of HSP, and thus, a delayed appearance of the rash may impact the correct diagnosis. Hardoff and Jaffe^31^ reported a 4-year-old child where the first scrotal swelling occurred 11 months before the HSP diagnosis. Before the diagnosis of HSP, 2 independent testicular swellings had occurred. When HSP cannot be diagnosed in a timely manner, the testicular examination should be included in the diagnostic and treatment procedures to raise.

Vasculitis is undoubted the cause of scrotal pain and swelling in HSP patients with scrotal involvement. According to recent studies, immune inflammation and oxidative stress play a vital role in the pathogenesis and progress of HSP.^32^ When immune inflammation occurs, vascular endothelial structure changes, neutrophil chemotactic aggregation, small arteries, veins, and human capillaries participate in the release of vasoactive substances, the permeability of blood vessels is increasing, the genitals are rich in blood flow, and the blood vessels of the scrotum are affected, such as swelling of the scrotum, etc. Leukocytes release cytokines, such as interleukin (IL)-6, IL-8, and IL-10, in the inflammatory state, leading to increased reactive oxygen species in the body. Inducing oxidative stress and excessive reactive oxygen species secretion will stimulate the secretion of inflammatory factors and expand the inflammatory response.^33^

Given the literature, scrotal involvement in HSP patients should be managed conservatively, with a short-term administration of steroid therapy and/or antibiotics rather than surgically. Treatment actively can also reduce the risk of other complications, such as nonsteroidal anti-inflammatory drugs for joint involvement. Corticosteroids, azathioprine, cyclophosphamide, and plasmapheresis are used for treating renal involvement or patients with inadequate response to conservative treatment. If the disease continues to develop and the skin rush recurs repeatedly, it is possible to remove the infection by surgical methods, such as caries repair and tonsillectomy. For cured patients, blood pressure and urine analysis should also be closely monitored to prevent HSP sequelae.^34^

For HSP with scrotal involvement other than testicular torsion, we found that the increase of d-dimer levels is also a risk factor. Increased levels of d-dimer is a sensitive marker of acute thrombosis.^35^ The deposition of immune complexes activates the body’s coagulation system, leaving the body in a hypercoagulable state, which can lead to genital ischemia, hypoxia, and tissue damage. Scrotal involvement, gastrointestinal symptoms, and elevated d-dimer are significantly associated with renal injury, and the presence of these factors increases the incidence of renal injury.^36^ The measurement of d-dimer has significant clinical value for patient diagnosis, treatment, and prognosis evaluation.

## CONCLUSIONS

Scrotal involvement is common in male patients with HSP. Clinical doctors should include early HSP in their differential diagnosis of rashes. Through careful physical examination and imaging examination, the clinician should be able to distinguish from testicular torsion. In an effort to prevent unnecessary surgery, conservative treatments are preferred. Additional attention needs to be paid to the relationship between d-dimer and scrotum involvement. As this study is limited by its narrative literature review and clinical case report as well as its small sample size, multicenter research is needed for HSP with scrotal involvement to define a diagnostic protocol, the best treatment strategy, and the prognosis.
